# Prevalence of mandibular, condylar and ramus asymmetry in panoramic radiographs of adult individuals. A cross-sectional study

**DOI:** 10.4317/jced.62144

**Published:** 2024-11-01

**Authors:** Sandra Pinto-Wong, Luis Ernesto Arriola-Guillén

**Affiliations:** 1 Orthodontic student, School of Dentistry, Universidad Científica del Sur, Lima, Perú; 2 Ph.D. and Associate Professor of the Division of Orthodontics, Universidad Científica del Sur, Lima, Perú

## Abstract

**Background:**

Mandibular asymmetry is more common than previously thought. The purpose of this study was to determine the prevalence of mandibular, condylar and ramus asymmetry by means of the Habets index using panoramic radiographs obtained from adult individuals.

**Material and Methods:**

This cross-sectional study evaluated 210 panoramic radiographs performed in adults attending a private referral radiology center in Lima, Peru. Radiographs of both genders were considered, including permanent dentition and of good quality. A trained and calibrated evaluator assessed mandibular, condyle and ramus height using the Habets method, considering asymmetry when the difference between the two sides was greater than 3%. Fisher’s exact test, the paired Student’s t-test and finally binary logistic regression were used to determine the characteristics of the asymmetries.

**Results:**

Mandibular asymmetry was present in 39.5%, condylar asymmetry in 81.4% and mandibular ramus asymmetry in 48.6%, with no differences between genders (*P*>0.05). Only women showed a difference between the two sides in mandibular (*P*=0.008), and condylar height (*P*=0.013), although multivariate analysis showed neither gender nor age to have any significant influence on the occurrence of mandibular, condylar or ramus asymmetries.

**Conclusions:**

The prevalence of mandibular, condylar and ramus asymmetries in the sample evaluated was significant, although most of these asymmetries can be considered mild, given that the highly sensitive Habets index classifies any difference greater than 3% as asymmetry. These asymmetries, although most of them could be clinically not very noticeable, should be considered when planning treatments. In addition, neither gender nor age was found to significantly influence the occurrence of these asymmetries.

** Key words:**Asymmetry, condyle, mandible, orthodontics.

## Introduction

Facial symmetry is the result of a balanced bone growth producing musculoskeletal structures, including the mandible, that are similar in size and shape on both sides of the face, taking into account a normal range of differences between the two sides that do not affect facial symmetry. Few perceptible asymmetries affect mandibular esthetics or function (1,2). Each side of the hemimandible is made up of the body, the ascending ramus, and the mandibular condyle, the individual or joint development of which may differ during the growth process (3).

Mandibular asymmetry is not rare and is usually diagnosed by a combination of studies, such as clinical evaluation, photographs, panoramic radiographs, posteroanterior cephalometry, and tomography (4-12). It has been shown that the lower third of the mandible is more prone to facial asymmetry, with the growth of the upper maxillary bone being sTable while the lower maxillary bone, which is the only mobile bone, is more sensitive to environmental factors (13).

The structures with the greatest growth potential in the mandible are the condylar cartilages. Injuries occurring to these cartilages during growth may alter their development, resulting in displacement of the lower jaw towards the affected side (4,5). Several risk factors have been described in relation to facial asymmetry beyond alterations of the facial growth pattern including gender, age, muscle activity, pathologies, congenital alterations, infections, trauma and occlusal interferences, and thus, the etiology of mandibular asymmetry is multifactorial (6-9).

At least two hypotheses have been generated to explain mandibular asymmetry; the first is that the asymmetries are simple morphological variations (14). The second hypothesis is that functional and mechanical variations induce asymmetry; i.e., masticatory forces give rise to joint loading over time, and these are related to the size of the condyle. Several studies have shown that the temporomandibular joint transfers masticatory forces from the mandible to the skull during mastication. This suggests that the magnitude of joint loads over time may be related to the size of the mandibular condyle. Humans tend to chew on one side of the mouth at a time, and most skulls, including juvenile specimens, show differential wear on either the right or left side of the dentition, suggesting that over a period of years more force is transmitted to one condyle or the other. The greater transmission of force through one condyle may be related to greater bone growth in that condyle, at least until the individual is fully developed. Therefore, it can be assumed that if there is a correlation between condylar asymmetry and which side of the dentition is more commonly worn, then there should also be a distinct positive or negative relationship between which side of the mouth has more worn teeth and which side has a larger condyle (14,15).

Regardless of the origin of the problem, orthodontists have developed various tools to diagnose problems related to mandibular asymmetry. In this regard, a widely used diagnostic tool is panoramic radiography, which is generally used to complement the clinical examination. This is a simple technique that provides low radiation doses and allows correct evaluation of the osseous structures (16,17). Likewise, several measurement indexes have been described in panoramic radiographs, the most reliable being those that measure vertically (16,18,19).

Previous studies evaluating mandibular asymmetry in different parts of the world have reported a prevalence of 23.9% and 12.5% in Caucasian men and women, respectively (20), 25% in Asians, (21,22), and 41.4% and 40.4% in European men and women, respectively (23). In Latin America, a study conducted in Chile revealed a statistically significant difference between the height of the condylar process and the left ramus of the mandible. The results showed that the height of the condylar process was greater in women than in men, with the greatest difference being observed in the mandibular ramus (24). Similarly, in Ecuador, the right ascending ramus sshowed a prevalence of greater height, which was more pronounced in men (25).

The Habets asymmetry index was described in 1988, and is considered to be one of the most widely used tools to compare the height of the ramus and condyle on both sides of the mandible to identify the presence of asymmetry with the formula: [(R-L)/(R+L)] x 100 % (26,27). While some individuals may present severe bony asymmetry, the frequency of mild asymmetry is greater in orthodontic practice. The aim of the present study was to determine the prevalence of mandibular, condylar and ramus asymmetry with the Habets index using panoramic radiographs obtained from adults.

## Material and Methods

This was a retrospective, cross-sectional study. It was approved by the Institutional Research Ethics Committee of the Universidad Científica del Sur with registration number POS-95-2024-00310. The study group consisted of 210 panoramic radiographs of adult individuals who attended a private referral radiology center in Lima, Peru between 2022 and 2023. The sample size was determined by means of a formula to estimate a proportion (the proportion of mandibular asymmetry) according to a pilot test performed in 100 panoramic radiographs, with the following data: Population: 600 panoramic radiographs, 95% confidence interval (CI), precision 5%, test power 80% and estimated proportion of mandibular asymmetry of 30%.

The following inclusion criteria were used: clearly observed digital panoramic radiographs, radiographs of males and females aged 18 to 50 years showing complete permanent dentition with or without the presence of third molars. Panoramic radiographs presenting distortion, ghost images, archival problems or damage, individuals with mixed dentition, or with orthodontic treatment, and also syndromic patients or individuals with evident tumors were excluded.

-Image acquisition

**Figure 1:**
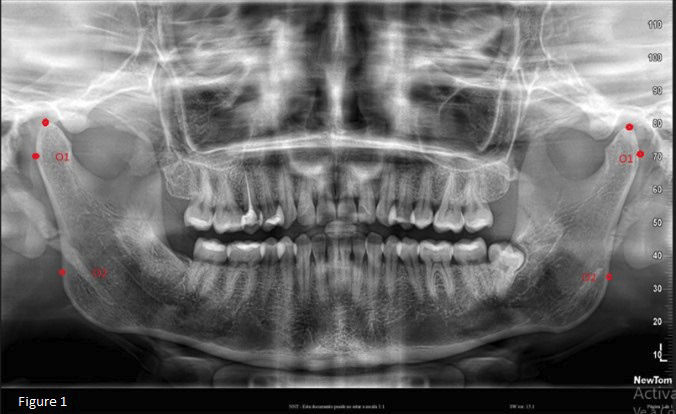
The most lateral point of the mandibular condyle (O1), the most lateral point of the mandibular ramus (O2), and the most superior point of the condyle.

Panoramic radiographs were extracted from the database of a private radiology center. Initially, the images were converted from PDF to JPG format. Subsequently, we identified and localized the relevant anatomical structures, the most lateral point of the mandibular condyle (O1) and the most lateral point of the mandibular ramus (O2). The points were then marked with the help of Tps Dig 2 version 2.32 software (Fig. 1).

**Figure 2:**
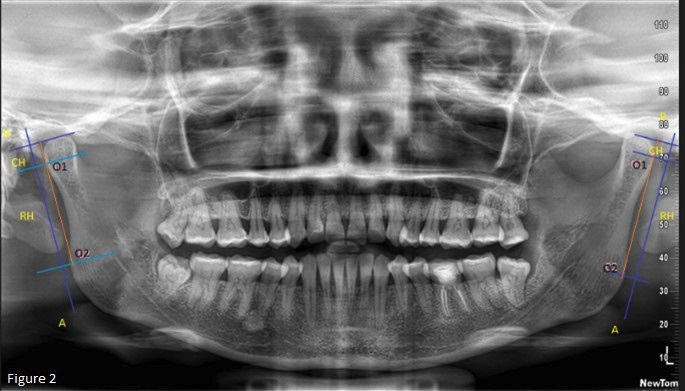
Line A, which represents a tangent line on the mandibular ramus, drawn from O1 to O2, and Line B, which is perpendicular from line A to the most superior area of the condyle. Condylar height (CH), defined as the distance from the point of intersection of line B with line A to point O1; ramus height (RH), determined as the distance between points O1 and O2 measured on line A; and CH and CH, which consisted of the sum of the two previous measurements.

After marking the points, lines were drawn, and measurements were made using Image J software. These lines were named Line A, which represents a tangent line on the mandibular ramus, drawn from O1 to O2, and Line B, which was perpendicular from Line A to the most superior area of the condyle. Using as reference the points and lines drawn, specific measurements were performed, including condylar height (CH), defined as the distance from the point of intersection of line B with line A to point O1; ramus height (RH), determined as the distance between points O1 and O2 measured on line A; and CH and RH, which consisted of the sum of the two previous measurements (Fig. 2).

Subsequently, the formula of the Habets index was applied to calculate mandibular RH, mandibular CH and CH plus RH as follows:

Condylar asymmetry index: (CHrhs-CHlhs/CHrhs+CHlhs)*100.

Ramus asymmetry index: (RHrhs-RHlhs/RHlhs+RHrhs)*100.

Condyle-and-ramus asymmetry index: [(CH+RHrhs) - (CH+RHlhs) / (CH+RHrhs) + (CH+RHlhs)*100.

(CH+RHlhs)] *100

The presence of asymmetry was considered when the difference was greater than 3%. The diagnosis was for each area evaluated, and asymmetry was considered when at least one index was altered.

-Calibration and training

A specialist orthodontist with more than 10 years of experience trained the main evaluator in the visualization and localization of the anatomical structures. Subsequently, the calibration between them was evaluated, and therefore all the measurements were taken at two separate times separated by one week, using the weighted Kappa and CCI test until values above 0.7 were obtained in all measurements.

-Statistical analysis

The data were processed and analyzed using IBM SPSS Statistics version 29 (IBM Corp., NY, USA). The prevalence of mandibular, condylar and ramus asymmetries were evaluated in the whole sample. Fisher’s exact test was then used to evaluate associations between asymmetries and gender. Subsequently, the lengths of both sides were compared using the paired Student’s t-test and, finally, binary logistic regression analysis was performed to evaluate the influence of predictor variables on the occurrence of asymmetries (*P*<0.05).

## Results

The initial characteristics of the sample are shown in  Table 1.  Table 2 shows the prevalence of mandibular, condylar and ramus asymmetry according to gender, finding that mandibular asymmetry is present in 34.3% of men and 42.0% of women, while condylar asymmetry is present in 83.6% of men and 80.4% of women and ramus asymmetry is present in 47.8% of men and 49.0% of women.  Table 3 shows the comparison of mandibular, condylar and ramus height between sides in men, with no significant difference being observed. On the other hand, a difference in the mandibular height of both sides was found in women (0.69 mm mean difference, *p*=0.008), CH (0.36 mm mean difference, *p*=0.013). Likewise, binary logistic regressions analysis showed the appearance of mandibular, condylar or ramus asymmetry considering gender and age, although without significant differences (*P*>0.05) ( Table 4).

## Discussion

In orthodontic clinical practice, the presence of bony asymmetry is not infrequent. In general, the prevalence of the most clinically evident cases is low and includes mandibular deviations, which should be treated with orthognathic surgery. However, the percentage of mild and to some extent compensable asymmetry is much higher in clinical practice. Nonetheless, the prevalence of asymmetry may be affected by genetic factors and be greater in determined ethnic groups. Orthodontists should be aware of these factors and take them into account for adequate treatment planning and management as well as the decision to perform maxillofacial procedures. For these reasons, the purpose of this research was to determine the prevalence of mandibular, condylar and ramus asymmetry in panoramic radiographs of Peruvian individuals.

Several imaging tests are useful for the diagnosis of mandibular asymmetry. Among these, panoramic radiography is a very useful initial method that allows comparative evaluation of the shape and size of the ramus, condyles, and mandibular bodies. In this sense, our results show a high prevalence of condylar asymmetry (83.6% in males and 80.4% in females, with no significant differences between the two genders). These values coincide with those reported in 2018 by Barreno and Macias (28), who obtained a prevalence of condylar asymmetry of 70.3%, in a sample of patients with definitive dentition. However, Kasimoglu *et al*. (27) obtained condylar asymmetry results of 3.5% and 9.49%, in Turkish adolescents. This variation in asymmetry was due to evaluation with a method that only includes severe asymmetries. There were also differences in the population samples and the variability in the ethnic and geographical characteristics of the populations studied may also influence the prevalence of condylar asymmetry. South American and Turkish populations may present different values due to cultural and genetic differences.

Regarding the mandibular ramus, the prevalence of asymmetry was 47.8% in men and 49.0% in women, also without significant differences between genders. These values are similar to the 38.7% described by Barreno and Macias (28). They also coincides with Galarza *et al*. (25) who found a high prevalence of mandibular ramus asymmetry, being greater in the right ramus and in men. The least prevalent asymmetry was that of condyle plus ramus height, which was found in 34.3% of men and 42.0% of women, which is a result some similar to that found by McCrea *et al*. (20) with a prevalence of mandibular asymmetry of 18%.

Additionally, although magnification of panoramic radiographs is not the most adequate method to perform linear measurements, comparisons between sides may be made to evaluate the presence of asymmetries, since the magnification is the same for both sides. In this sense, in our study no significant difference was found in the comparison of measurements in men. On the contrary, women showed a significant difference in mandibular height and CH, but despite being significant, the difference was small and should therefore be analyzed with caution. Additionally, we evaluated the influence of some predictor variables (gender, age) on the appearance of mandibular, condylar or ramus asymmetry by means of logistic regression, but found no significant results. Likewise, Alfaro *et al*. (29) showed that gender is not a conditioning factor of vertical mandibular asymmetry, and the values reported in the study of Barreno and Macias (28) showed no statistically significant relationship with the age or gender of the patients.

Finally, the results of the present study have important implications for the diagnosis and treatment of mandibular asymmetry and should be taken into account for treatment planning and management of patients in orthodontics and oral and maxillofacial surgery.

## Conclusions

1. The prevalence of mandibular, condylar and ramus asymmetries was 39.5%, 81.4% and 48.6% respectively in the sample evaluated. These values are considerable and should be taken into account by clinicians for treatment planning. Gender and age do not influence the appearance of these asymmetries. It is important to note that the Habets index is very sensitive for the detection of any minimal deviation greater than 3% and it is possible that the high prevalence of condylar asymmetry involves mainly mild asymmetries. This means that they may not be clinically relevant and do not necessarily imply mandibular pathology or dysfunction.

2. For future studies, the use of a higher threshold, such as 8%, is recommended to identify more clinically significant asymmetries. This would allow better differentiation between mild and severe asymmetries, facilitating the identification of cases with potential pathologic relevance and helping to establish a more accurate understanding of the prevalence and impact of condylar asymmetries.

## Figures and Tables

**Table 1:** Initial characteristics of the sample.

Sex	n	Mean	SD
Male	67	30.31	6.924
Female	143	29.84	8.031

*P*=0.678 Student’s t-test SD: standard deviation

**Table 2:** Prevalence of mandibular, condylar and ramus asymmetry in general and according to gender.

Sex	Mandibular asymmetry				p
	Indicator	Absent	Present	Total	
Male	n	44	23	67	0.364
	%	65.7	34.3	100	
Female	n	83	60	143	
	%	58.0	42	100	
Total	n	127	83	210	
	%	60.5	39.5	100	
Sex		Condylar asymmetry		Total	p
	Indicator	Absent	Present		
Male	n	11	56	67	0.704
	%	16.4	83.6%	100	
Female	n	28	115	143	
	%	19.6	80.4%	100	
Total	n	39	171	210	
	%	18.6	81.4%	100	
Sex		Ramus asymmetry		Total	p
	Indicator	Absent	Present		
Male	n	35	32	67	0.883
	%	52.2	47.8	100	
Female	n	73	70	143	
	%	51	49	100	
Total	n	108	102	210	
	%	51.4	48.6	100	

Fisher’s Exact Test

**Table 3:** Comparison of mandibular, condylar and ramus height between mandible sides according to gender.

Sex	Measurement	n	Mean	SD	Mean difference	p
Male	Mandibular height right	67	49.10	4.10	-0.21	0.624
	Left mandibular height	67	49.31	4.61		
	Right condyle height	67	6.81	1.73	-0.01	0.953
	Left condyle height	67	6.82	1.71		
	Right ramus height	67	42.29	4.25	-0.11	0.827
	Height of left ramus	67	42.41	4.75		
Female	Mandibular height right	143	44.40	3.99	-0.69	0.008*
	Left mandibular height	143	45.10	4.24		
	Right condyle height	143	7.33	1.80	-0.36	0.013*
	Left condyle height	143	7.69	2.04		
	Right ramus height	143	37.07	3.49	-0.29	0.263
	Height of left ramus	143	37.37	4.04		

* Significant, paired t-test SD: standard deviation

**Table 4:** Binary logistic regression to determine the occurrence of mandibular, condylar or ramus asymmetry according to predictor variables.

Predictor variables	p	Exp. (B)	95% CI for EXP(B)	
			Inferior	Superior
Mandibular asymmetry				
Female	---	---	---	---
Male	0.294	1.38	0.76	2.53
Age	0.933	1.00	0.96	1.04
Condylar asymmetry				
Female	---	---	---	---
Male	0.616	0.82	0.38	1.77
Age	0.215	1.03	0.98	1.08
Ramus asymmetry				
Female	---	---	---	---
Male	0.880	1.05	0.58	1.87
Age	0.749	0.99	0.96	1.03

CI: confidence interval

## Data Availability

The datasets used and/or analyzed during the current study are available from the corresponding author.
